# The efficacy of two rotary NiTi instruments and H-files to remove gutta-percha from root canals

**DOI:** 10.4317/medoral.17582

**Published:** 2011-12-06

**Authors:** Kerem E. Akpınar, Demet Altunbaş, Alper Kuştarcı

**Affiliations:** 1 DDS, PhD, Assistant Professor, Department of Endodontics, Faculty of Dentistry, Cumhuriyet University, Sivas, Turkey; 2 DDS, PhD, Research assistant, Department of Endodontics, Faculty of Dentistry, Cumhuriyet University, Sivas, Turkey

## Abstract

Objective: The aim of this study was to evaluate the efficacy of R-Endo® and K3® rotary nickel-titanium instruments compared with manual instrumentation with H-files, with use of a solvent, for removal of gutta-percha during retreatment. Study design: Forty five freshly extracted human single-rooted teeth, each with one root canal, were instrumented with K-files and filled using cold lateral compaction of gutta-percha and AH 26® sealer. The teeth were randomly divided into three groups of 15 specimens each. Removal of gutta-percha was performed with the following devices and techniques: Group 1 (H-files), Group 2 (R-Endo®), and Group 3 (K3®). The specimens were rendered transparent for the evaluation of the area of remaining gutta-percha/sealer in buccolingual and mesiodistal directions. Statistical analysis as performed by using one-way ANOVA and Kruskal-Wallis tests (p=0.05). Results: All retreatment techniques used in this study left some filling material inside the root canal. Images in buccolingual and mesiodistal directions showed no significant differences between the groups (p>0.05). Conclusions: Under the experimental conditions, the remaining filling material after retreatment was similar for each group.

** Key words:**Gutta-percha removal, K3®, NiTi, R-Endo®.

## Introduction

Nonsurgical endodontic retreatment is an attempt to re-establish healthy periapical tissues after inefficient treatment or reinfection of filled root canals because of coronal or apical leakage. It requires regaining access to the entire root canal system through removal of the root canal filling materials, further cleaning, and refilling (1). The successful removal of gutta-percha and sealer is an important step; however, it has not been proved that the complete removal of root filling materials will ensure success of root canal retreatment and that remaining material will cause the retreatment to fail. Nevertheless, removing the maximum amount of filling material from inadequately prepared and/or filled root canal systems appears to be essential in order to uncover remaining necrotic tissue or bacteria that may be responsible for the persistent disease and enable thorough chemomechanical reinstrumentation and redisinfection of the root canal system (2).

Although, numerous materials have been described for root canal filling, gutta-percha in combination with a sealer is the most frequently used. Many techniques have been described for the removal of filling material from the root canal system including rotary instruments, ultrasonic instruments, heat, hand files combined with heat or chemicals such as solvents (3). Conventionally, the removal of gutta-percha using manual files with or without solvent can be a tedious, time-consuming process, especially when the root filling material is well condensed (4). Therefore, rotary nickel-titanium (NiTi) instruments have been used for the removal of filling materials from root canal walls, and various studies reported their efficacy, cleaning ability and safety (5-7).

The R-Endo® (Micro-Mega, Besançon, France) instrumentation system, specifically dedicated to retreatment procedures, has been developed in 2003. The system is composed of four instruments: Re (size 25, 0.12 taper) to flare the first few millimetres of the canal, and three files R1, R2 and R3 dedicated to each root canal third to a size 25, with 0.08, 0.06 or 0.04 tapers respectively. An optional finishing file Rs (size 30, 0.04 taper) is available if required. The files have a triangular crosssection with three equally spaced cutting edges and no radial land; the tip of the files is claimed to be inactive (3).

The K3® file (SybronEndo, West Collins, CA, USA) is a rotary instrument with a radial land relief in combination with a positive rake angle, a flattened noncutting tip, and an asymmetrical constant tapered active file design with variable helical flute and variable core diameter. These are features that are claimed to enhance cutting-efficiency, debris removal, and file guidance and strength (8).

The aim of this in vitro investigation was to evaluate the efficacy of two rotary NiTi instruments (K3® and R-Endo®) removal of gutta-percha during root canal retreatment compared with manual instrumentation using H-files.

## Material and Methods

-Specimen preparation

In the present study, forty five freshly extracted human maxillary central incisors were used. All teeth were analyzed with digital radiographs (Schick Tech. Inc., Long Island City, NY, USA) in buccal and proximal directions to check for a single canal. Each tooth included the following criteria: curvatures between 0-5° (9), mature root, and absence of root filling, resorption, or calcifications. Soft tissue and calculus were mechanically removed from the root surfaces and the teeth were stored in physiological saline solution until required.

Access cavities were prepared using high-speed diamond burs with water cooling. Apical patencies were determined with a size 10 K-file (Dentsply Maillefer, Ballaigues, Switzerland). The working length was established 1 mm short of the apical foramen. The coronal portion of the canal was flared with sizes 2-3 Gates-Glidden burs (Dentsply Maillefer, Ballaigues, Switzerland). The tooth was then further prepared with a step-back technique using K-files apically to a master apical file size 30 and coronally to a file size 55. Each instrument was used only for the preparation of three teeth. After each instrument was used and before proceeding to the next, canals were irrigated with 2 ml of 2.5% NaOCl. When instrumentation of the root canal was complete, 17% EDTA was applied for 1 min to remove the smear layer, and the canal was flushed again with 2.5% NaOCl. The root canals were then dried with absorbent paper points.

-Root filling

The root canals were filled with cold lateral condensation (CLC) of gutta-percha and root canal sealer AH 26® (Dentsply De Trey, Konstanz, Germany) by one operator. A master gutta-percha cone size 30 was selected and tug-back was checked. AH 26® sealer was mixed according to the manufacturer’s instructions and the master cone was coated with sealer and positioned into the canal. Thereafter, accessory gutta-percha cones sizes 20 and 25 were laterally compacted using finger spreaders (Dentsply Maillefer, Ballaigues, Switzerland), until they could not be introduced more than 3 mm into the canal. A heated plugger was used to remove 2 mm of the gutta-percha coronally. The root canal filling was compacted vertically with a cold plugger. The roots were radiographed in buccolingual and mesiodistal directions to confirm adequacy of the root filling. Regardless of tooth length, the extent of the root filling was uniformly limited to 16 mm from the apex by sectioning the coronal surplus using a heatened hand plugger so that the volume of the gutta-percha filling was approximately equal for all teeth. Then the access cavities were filled temporarily (Cavit®, Espe, Seefeld, Germany). All teeth were stored in a humidor at 37°C and 100% humidity for 2 weeks to allow complete setting of the sealer.

-Retreatment procedures

The teeth were randomly divided for retreatment into three groups of 15 specimens each. After the temporary filling materials were removed from the access cavities, 0.1 ml of eucalyptol was first placed for 3 minutes into the access cavity to soften the root filling material. Then two or three additional drops were applied if needed with a maximum of 0.2 ml per canal. All rotary instruments were used at a constant speed of 300 rpm and torque recommended by the manufacturers.

Group 1 (H-files): The canal was reinstrumented with H-files (Dentsply Maillefer) in sizes 20, 25, and 30 in a circumferential quarter-turn push-pull filing motion to remove gutta-percha and sealer from the canal. A step-back procedure with H-files was then completed coronally in 1 mm increments to file size 55.

Group 2 (R-Endo®): R-Endo® instruments (Micro-Mega) were used with an inget type contra-angle handpiece (Inget® 06 contra-angle; Micro-Mega) and manipulated in a gentle in-and-out motion according to the manufacturer’s instructions. A manual file was used first to relocate the canal orifices, then the Re instrument removed the first 2-3 mm of the filling. R1 and R2 instruments were used to one-third and two-thirds of the estimated working length respectively. R3 instruments was used at the working length with circumferential filing action. Finally, the retreatment procedure was concluded with the use of Rs instrument at the working length.

Group 3 (K3®): K3® (SybronEndo) instruments were used in a crown-down manner according to manufacturer’s instructions using a gentle in-and-out motion. Instruments were withdrawn when resistance was felt and changed for the next instrument. File sequences were as follows: size .06/25 was used at one-half of the working length; size .06/20 was used between one-half and two-thirds of working length; and instruments of sizes .04/20, .04/25, and .04/30 were used to the working length.

To standardize procedures throughout the study, only one operator conducted the experiments to avoid variables during specimen preparation. All instruments were used for a maximum of three root canals and then discarded. Also, any deformed instruments were discarded. During retreatment, root canals were constantly irrigated with 2.5% NaOCl. The irrigant was delivered by disposible plastic syringe with an attached 27-gauge stainless steel needle that was placed down the canal until slight resistance was felt. Gutta-percha removal was judged complete when the working length was reached and no more gutta-percha could be removed with the instruments used.

-Evaluation of canal wall cleanliness

**Figure 1:**
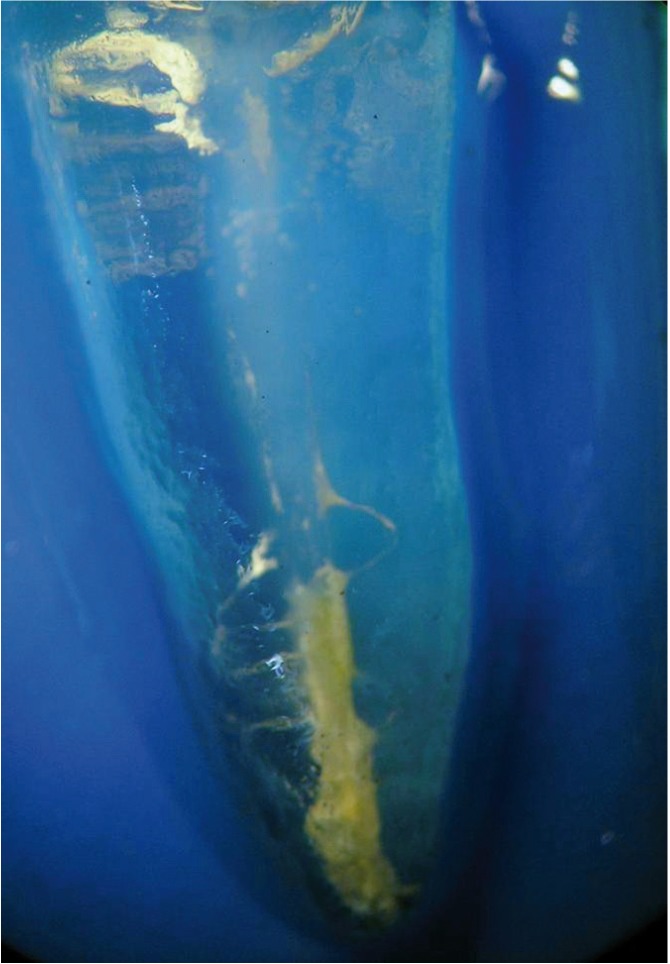
Gutta-percha and sealer remnants on the root canal walls.

**Figure 2:**
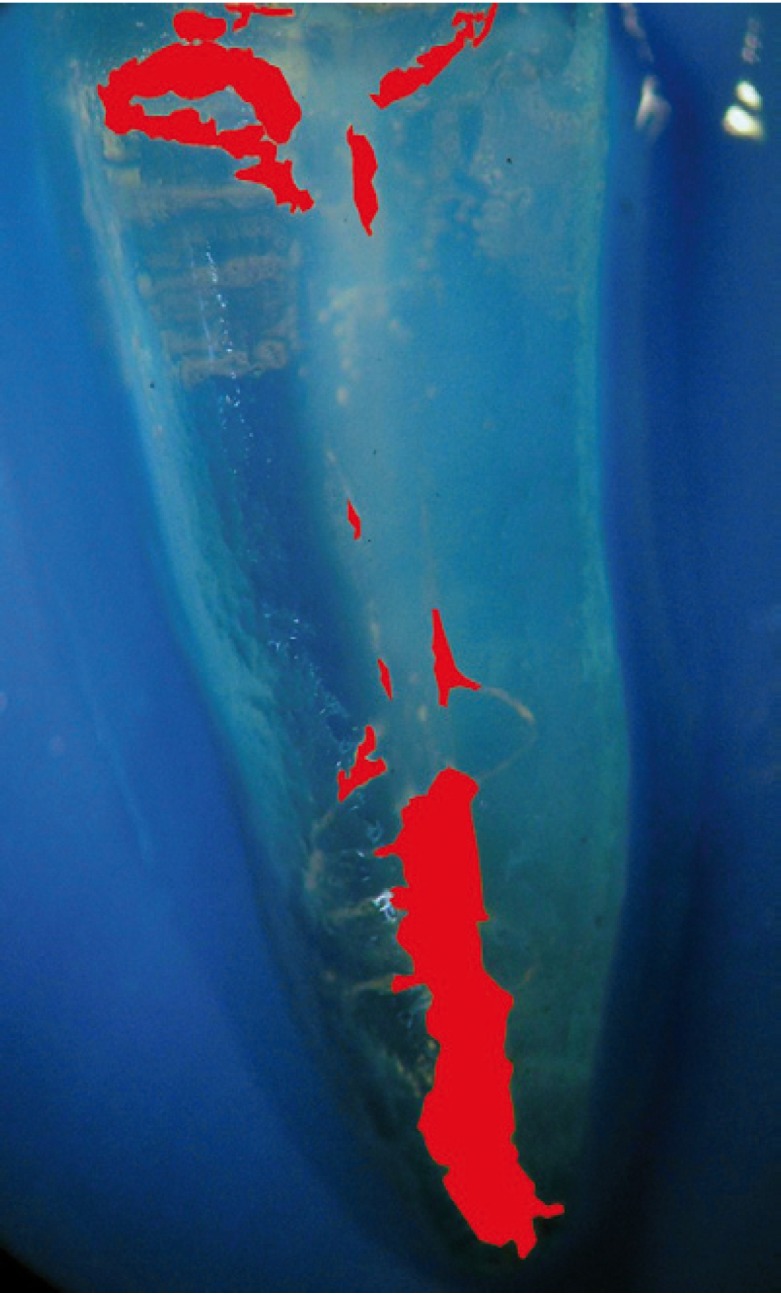
Calculation of the area of gutta-percha and sealer using imaging programme after cleared specimen.

The teeth were rendered transparent according to the technique described by Robertson et al. (10). They were demineralized in 5% nitric acid for 72 h at room temperature, dehydrated in ascending concentrations of ethanol (80%, 90%, 96%), and stored in methyl salicylate at room temperature until they became clear. The specimens were then photographed in a microscope with a digital camera at 6X magnification, and gutta-percha/sealer on the canal walls was measured in mm2 using image analysis software (ImageJ®, U.S. National Institutes of Health, Bethesda, MD, USA) in the buccolingual and mesiodistal directions (Figs. 1,2).

-Statistical analysis

The results of the study were evaluated statistically using one-way ANOVA and Kruskal-Wallis tests. P values were computed and compared with statistical significance at the level of p=0.05.

## Results

All retreatment techniques used in this study left some filling material inside the root canal ( Table 1). Images in buccolingual and mesiodistal directions showed no significant differences between the groups (p>0.05). The mean values of remaining filling material of the three groups, respectively, from lowest to highest, were Group 3 (K3®), Group 2 (R-Endo®) and Group 1 (H-files), imaged in both directions.

**Table 1:**
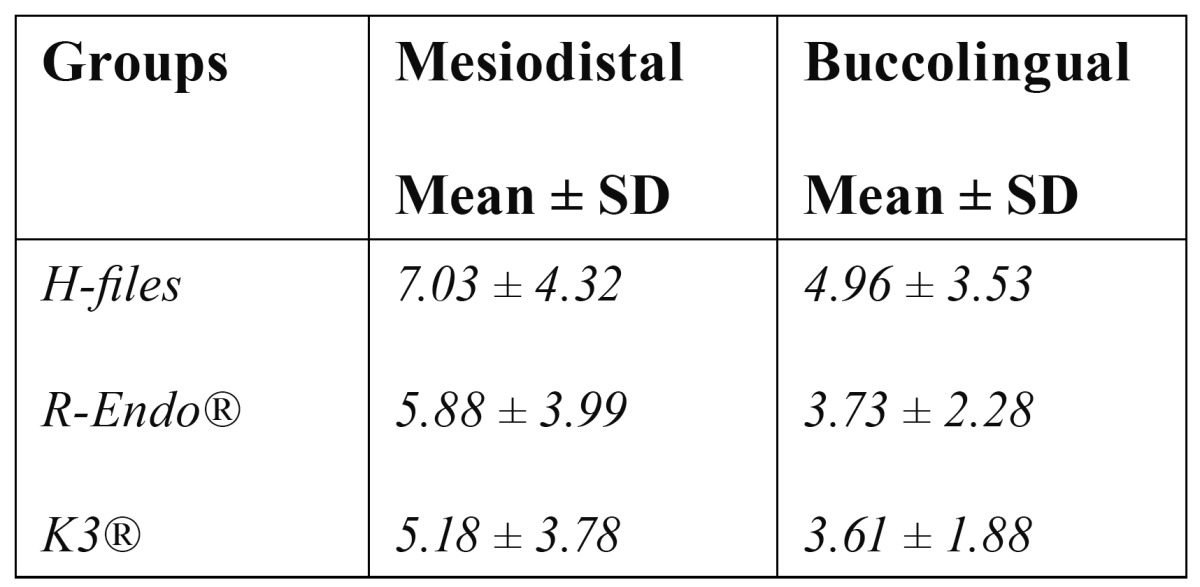
The amount of remaining filling material (mm2).

## Discussion

In the present study, one of the most difficult to control parameters was the extent of the anatomical variations that are generally present in human teeth. Variations in original root canal morphology greatly influence the changes that occur after root canal preparation and as a logical extension, after retreatment procedures (11). In order to minimize these variables a standardized length of root canal filling was adhered to and only teeth with straight canals in both buccolingual and mesiodistal directions were selected.

The root canals were filled using the CLC before retreatment, in the present study. This obturation technique was used in many retreatment studies (5,12-14). In earlier retreatment studies remaining gutta-percha was assessed radiographically (15) or roots were split longitudinally and residual gutta-percha and sealer were measured linearly (5) or with evaluation scales: e.g., severe, moderate, mild or no retreatment debris (13,14). More recently, the micro CT scanner has been used (16,17) for this purpose. Ideally, three-dimensional visualization of the root canal system would provide a better understanding of the distribution of the debris after retreatment (15). Schirrmeister et al. (18) reported that residual material might be lost by splitting the roots longitudinally. Hence, the roots were cleared to allow measurement of the area of remaining gutta-percha/sealer, in the present study.

Chloroform is known to be most efficient in dissolving gutta-percha (19). However, it has been reported to be locally toxic in contact with periradicular tissues, to be hepatotoxic and nephrotoxic and has been classified as a carcinogen (20,21). Eucalyptol has been reported to be a safe and efficient noncarcinogenic alternative to chloroform (22). Therefore, it was used as a solvent, in the present study.

Some studies have revealed the superiority of rotary NiTi instrumentation over manual instrumentation in terms of the amount of remaining filling material (6,7,12). In contrast, studies observed similar amounts of residual root filling material and sealer after rotary NiTi and manual instrumentation in straight and curved root canals (15,23), while some studies have reported the superiority of manual instrumentation over rotary NiTi instrumentation (24,25). The present study found no significant differences between the tested groups. Review of the literature revealed that some studies had investigated the effectiveness of K3® instruments in the removal of gutta-percha during endodontic retreatment. Masiero and Barletta (26) evaluated various techniques for removing gutta-percha from root canals using K-type files, M4® system with K-type files, and Endo-gripper® with K-type files in comparison with K3® instrument, and concluded that there were no significant differences between these methods of removal when the entire canal was evaluated. However, Saad et al. (7) and de Carvalho Maciel and Zaccaro Scelza (25) reported that the mean ratio of remaining filling material in the entire canal was less with the K3® than with hand instruments, and the difference was statistically significant. This discrepancy with both previous studies could be explained with differences in retreatment methods (apical enlargement, taper and size of instruments). Saad et al. (7) used K3® instruments in the following sequence: Size 25 (0.10 taper), size 25 (0.08 taper), and size 20 (0.06 taper) in a crown-down technique to remove the gutta-percha until the working length was reached. Completion of gutta-percha removal and cleaning of canal walls was done using size 25 (0.06 taper) followed by size 30 (0.06 taper) to the working length. Because of using the greater taper of K3® instruments might be a reason for the effective gutta-percha removal in that study. de Carvalho Maciel and Zaccaro Scelza (25) used K3® 0.04 taper instruments of sizes 60, 50 and 45 sequentially to reach working length. The apical diameter was also enlarged to a size 45 at working length. Difference may be attributed to the fact that in that study, the K3® instruments that were used to remove the gutta-percha were three sizes larger than the master apical file, compared to using the same final retreatment K3® instrument size as the master apical file size in the present study. In contrast, Bueno et al. (27) found that K3® instruments were less effective in removing filling material from root canal walls than manual instruments in straight root canals. The contrast between the outcome of that previous study and the present study may be explained by use of K-files plus H-files to reach the working length. Also, they stated that the design of the flutes of the H-files facilitates gutta-percha removal (27). Therefore, H-files might be remove gutta-percha in large pieces, leaving remaining material of such a small size.

According to the manufacturer, R-Endo® instruments are specially designed to be used in retreatment. In earlier studies, Taşdemir et al. (28) and Fenoul et al. (3) reported that R-Endo® retreatment files and H-files have similar effectiveness in removing filling material in straight root canals. Similarly, Gergi and Sabbagh (29), in curved root canals, reported no significant difference between R-Endo® retreatment and H-files. Our results were similar to these previous studies that R-Endo® retreatment instruments and H-files showed no significant difference. However, Unal et al. (24) found that R-Endo® retreatment files were less effective in removing filling material from root canal walls than manual instruments. This might be due to the fact that K-files were used in combination with H-files to remove the gutta-percha mass in this previous study and this combination may have advantages.

No instrument fractures occurred during gutta-percha removal. The speed of the rotary NiTi instruments was adjusted according to the manufacturer’s recommendation. The low-torque handpiece approved to increase tactile sensitivity, give better control of rotary instrumentation, and reduced the risk of instrument fracture (30). Also, using each set of instruments to prepare a maximum of three root canals, plus the use of eucalyptol as a solvent, might be an additional reason for the lack of instrument fracture in this study.

## Conclusions

The current findings indicate that all instruments used in retreatment, rotary NiTi or hand, left some filling material inside the root canal. Also, no significant differences were found between all tested instruments.
